# Hydrogen Sulfide Downregulates Oncostatin M Expression via PI3K/Akt/NF-κB Signaling Processes in Neutrophil-like Differentiated HL-60 Cells

**DOI:** 10.3390/antiox12020417

**Published:** 2023-02-08

**Authors:** Na-Ra Han, Seong-Gyu Ko, Hi-Joon Park, Phil-Dong Moon

**Affiliations:** 1 College of Korean Medicine, Kyung Hee University, Seoul 02447, Republic of Korea; 2 Korean Medicine-Based Drug Repositioning Cancer Research Center, College of Korean Medicine, Kyung Hee University, Seoul 02447, Republic of Korea; 3 Department of Preventive Medicine, College of Korean Medicine, Kyung Hee University, Seoul 02447, Republic of Korea; 4 Department of Anatomy & Information Sciences, College of Korean Medicine, Kyung Hee University, Seoul 02447, Republic of Korea; 5 Center for Converging Humanities, Kyung Hee University, Seoul 02447, Republic of Korea

**Keywords:** oncostatin M, hydrogen sulfide, neutrophil-like differentiated HL-60 cells, phosphatidylinositol 3-kinase, Akt, nuclear factor-κB

## Abstract

The cytokine oncostatin M (OSM) is regarded as a critical mediator in various inflammatory responses. While the gaseous signaling molecule hydrogen sulfide (H_2_S) plays a role in a variety of pathophysiological conditions, such as hypertension, inflammatory pain, osteoarthritis, ischemic stroke, oxidative stress, retinal degeneration, and inflammatory responses, the underlying mechanism of H_2_S action on OSM expression in neutrophils needs to be clarified. In this work, we studied how H_2_S reduces OSM expression in neutrophil-like differentiated (d)HL-60 cells. To evaluate the effects of H_2_S, sodium hydrosulfide (NaHS, a donor that produces H_2_S), ELISA, real-time PCR (qPCR), immunoblotting, and immunofluorescence staining were utilized. Although exposure to granulocyte–macrophage colony-stimulating factor (GM-CSF) resulted in upregulated levels of production and mRNA expression of OSM, these upregulated levels were reduced by pretreatment with NaHS in dHL-60 cells. Similarly, the same pretreatment lowered phosphorylated levels of phosphatidylinositol 3-kinase, Akt, and nuclear factor-kB that had been elevated by stimulation with GM-CSF. Overall, our results indicated that H_2_S could be a therapeutic agent for inflammatory disorders via suppression of OSM.

## 1. Introduction

Hydrogen sulfide (H_2_S) is a gaseous signaling molecule that has a role in numerous pathophysiological conditions, including hypertension, inflammatory pain, osteoarthritis, ischemic stroke, oxidative stress, retinal degeneration, and inflammatory responses [1,2,3,4,5,6]. Our previous work also demonstrated that sodium hydrosulfide (NaHS) inhibited inflammatory cytokine TSLP levels and improved immune function [7,8]. However, the regulatory mechanism of NaHS on oncostatin M (OSM) expression has not been determined. Thus, in this work, we examined how NaHS regulates OSM expression in neutrophil-like differentiated (d)HL-60 cells.

Because of its elevated levels in patients with cancer, OSM is regarded as a cancer-related cytokine [9,10]. More broadly, OSM is regarded as a proinflammatory cytokine and is produced by a wide range of cells, such as macrophages, dendritic cells, activated T cells, monocytes, and neutrophils [11,12,13,14,15]. It is known that OSM plays a role in a variety of physiologic and pathologic conditions, including the growth regulation of cancers, reconstruction of the extracellular matrix, hematogenesis, liver regeneration, cardiac reconstruction, and inflammatory responses [10,16,17,18,19]; further, it is key in diverse inflammatory conditions [10]. The proinflammatory cytokine OSM is involved in inflammatory reactions in arthritic as well as hepatic disorders [10,20] and is implicated in respiratory inflammatory diseases, including rhinitis and asthma [21,22]. It has been reported that augmented inflammatory reactions have resulted from exposure to recombinant human OSM in normal human intestinal cells [11], and in human HaCaT keratinocytes, stimulation with recombinant human OSM has led to increased inflammatory responses [15]. In our previous work, stimulation with recombinant human OSM led to increased IL-1β secretion in human HaCaT keratinocytes, denoting the contribution of OSM to inflammatory responses [23]. Pothoven et al. [22] revealed that the main sources of OSM in pulmonary inflammatory disorders are neutrophil cells. There is no study that suggests the mechanisms of OSM regulation by NaHS in neutrophil cells. We thus researched how NaHS regulates OSM production and expression in dHL-60 cells. 

Generally, phosphatidylinositol 3-kinase (PI3K) is regarded as an important factor in the modulation of various intracellular signaling cascades [24]. Akt, which is known as a downstream kinase of PI3K, plays an essential role in inflammatory responses [25]. Thus, PI3K/Akt signaling processes play a pivotal role in the modulation of the cytokine system [26]. It has been reported that the PI3K/Akt pathway is critical in a wide range of disorders from tumors to heart disorders and inflammatory disorders [27]. Akt activation results in nuclear factor (NF)-κB (i.e., a downstream factor of Akt) activation [26]. It is known that NF-κB also plays a pivotal role in inflammatory reactions [28], and research has shown that OSM production and expression are controlled by PI3K/Akt/NF-κB signal cascade in osteoblast cells [28]. In the current work, we studied how NaHS reduces OSM expression in dHL-60 cells. 

## 2. Materials and Methods

### 2.1. Materials

NaHS was prepared by Samchun Pure Chemical Co., Ltd. (Pyeongtaek, Gyeonggi, Republic of Korea). We obtained all the antibodies for ELISA from R&D Systems (Minneapolis, MN, USA), phosphorylated (p)-PI3K p85 from Cell Signaling Technology (Danvers, MA, USA), and most of the antibodies for Western blot analysis from Santa Cruz Biotechnology (Santa Cruz, CA, USA).

### 2.2. Cell Culture

For the HL-60 cell culture, RPMI 1640 (Gibco, Grand Island, NY, USA) containing 10% FBS was used. For differentiation, HL-60 cells were exposed to 1.3% DMSO for 7 days. Neutrophil markers (CD11b and TERT) were checked (Figure S1). Human GM-CSF (5 ng/mL) was utilized to activate the cells, according to previous work [23,29].

### 2.3. Cell Viability 

NaHS or PBS was pretreated in dHL-60 cells (1 × 10^5^/mL) for 1 h, and GM-CSF (5 ng/mL) was treated for 4 h. Cell viability was measured, as previously described [30,31,32,33,34,35].

### 2.4. OSM Measurement 

NaHS or PBS was pretreated in dHL-60 cells (5 × 10^5^/mL) for 1 h, and GM-CSF (5 ng/mL) was treated for 4 h. OSM production was assessed using ELISA, as detailed elsewhere [36,37,38,39]. 

### 2.5. Real-Time Quantitative PCR

NaHS or PBS was pretreated in dHL-60 cells (1 × 10^6^/mL) for 1 h, and GM-CSF (5 ng/mL) was treated for 30 min. Real-time quantitative PCR was performed, as already described [40,41].

### 2.6. Western Blot Analysis 

NaHS or PBS was pretreated in dHL-60 cells (5 × 10^6^/mL) for 1 h, and GM-CSF (5 ng/mL) was treated for the indicated time (15 min for PI3K, 30 min for Akt, and 30 min for NF-κB). Western blot analysis was conducted, as already reported [42,43,44,45,46,47,48]. 

### 2.7. Immunofluorescence Analysis 

NaHS or PBS was pretreated in dHL-60 cells (1 × 10^6^/mL) for 1 h, and GM-CSF (5 ng/mL) was treated for 30 min. Immunofluorescence analysis was conducted, as per the literature [49,50].

### 2.8. Statistical Analysis

The data were analyzed by one-way analysis of variance (ANOVA) with Tukey’s post hoc test as well as an independent t-test for statistical analysis (IBM SPSS Statistics version 25, Armonk, NY, USA). *p* < 0.05 was considered to indicate statistical significance.

## 3. Results

### 3.1. NaHS Represses OSM Secretion in dHL-60 Cells 

**Figure 1:**
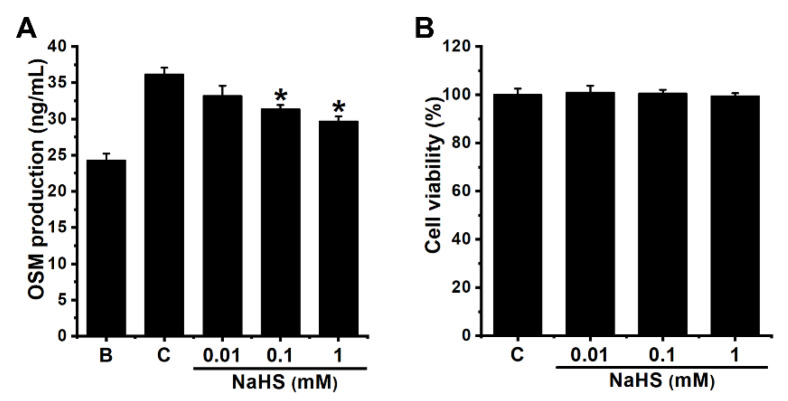
Inhibition of OSM production by NaHS in dHL-60 cells. (**A**) Cells (5 × 10^5^/mL) were pretreated with NaHS (0.01 to 1 mM) for 1 h, followed by GM-CSF-stimulation (5 ng/mL) for 4 h. (**B**) Cell viability was assessed by means of an MTT assay. Blank (B) corresponds to PBS treated cells without GM-CSF stimulation, and control (C) corresponds to PBS treated cells stimulated by GM-CSF. Results are expressed as the mean ± SEM from the three separate experiments. * *p* < 0.05, as compared with the PBS treated cells stimulated by GM-CSF.

First of all, we examined whether NaHS repressed OSM secretion in dHL-60 cells, since the main source of OSM in pulmonary inflammatory disorders is the neutrophil cell. Since OSM levels reached maximum production 4 h after GM-CSF stimulation [23], NaHS was pretreated in dHL-60 cells for 1 h and then GM-CSF was treated for 4 h. Upregulated OSM production, which resulted from exposure to GM-CSF, was similar to that of our earlier findings [23]. Pretreatment with NaHS induced downregulation of OSM production in dHL-60 cells (Figure 1A). NaHS (0.01 to 1 mM) treatments showed decreasing OSM levels (i.e., 33.167 ± 1.443, 31.333 ± 0.600, and 29.667 ± 0.704, respectively), while those of the control and blank groups were 36.150 ± 0.955 and 24.267 ± 0.966, respectively. Exposure to NaHS did not affect cell viability (Figure 1B). In addition, OSM production levels did not increase early on (15 min and 30 min after GM-CSF stimulation, Figure S2A). To have a convincing result that NaHS can regulate the expression of OSM, we used mast cell—an important cell in inflammatory disorders—line HMC-1 cells. Stimulation of HMC-1 cells with phorbol myristate acetate (PMA) plus calcium ionophore A23187 did not result in an increase in OSM levels (Figure S2B).

### 3.2. NaHS Decreases OSM mRNA Expression in dHL-60 Cells

**Figure 2:**
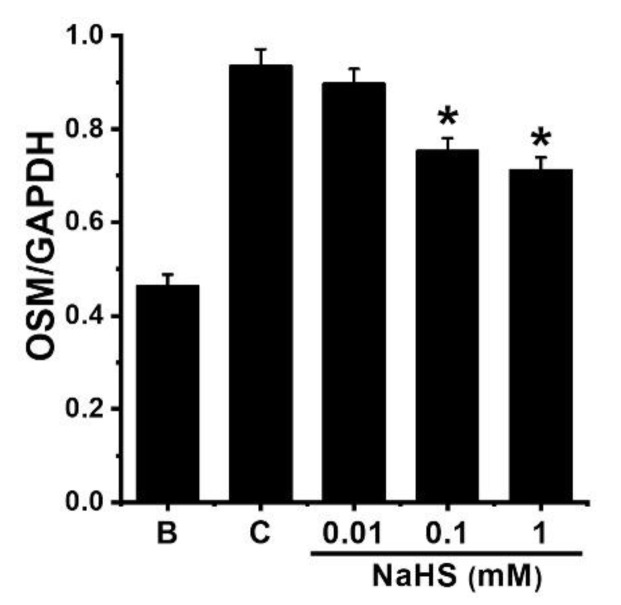
Inhibition of OSM mRNA expression by NaHS in dHL-60 cells. Cells (1 × 10^6^/mL) were pretreated with NaHS (0.01 to 1 mM) for 1 h, followed by GM-CSF-stimulation (5 ng/mL) for 30 min. Blank (B) corresponds to PBS treated cells without GM-CSF stimulation, and control (C) corresponds to PBS treated cells stimulated by GM-CSF. Results are expressed as the mean ± SEM from the three separate experiments. * *p* < 0.05, as compared with the PBS treated cells stimulated by GM-CSF.

To investigate whether NaHS could inhibit mRNA expression of OSM, NaHS was pretreated in dHL-60 cells for 1 h and then GM-CSF was treated for 30 min. As shown in the previous work [23], stimulation with GM-CSF led to increased mRNA expression of OSM (Figure 2). However, this increased mRNA expression was lowered by pretreatment with NaHS (Figure 2). Pretreatments with NaHS (0.01 to 1 mM) showed reduced OSM mRNA levels (i.e., 0.897 ± 0.032, 0.753 ± 0.028, and 0.712 ± 0.027, respectively). The control and blank groups showed 0.935 ± 0.037 and 0.463 ± 0.025, respectively. Co-treatment of NaHS with a PI3K inhibitor (wortmannin) as well as NaHS with a NF-κB inhibitor (PDTC) showed a synergy effect in the suppression of OSM mRNA expression in dHL-60 cells, but was not seen with Akt inhibitor (Figure S3). We investigated the modulatory effect of 1 mM of NaHS in the ensuing experiments (Western blot analysis and immunofluorescence staining), since the effect of 1 mM of NaHS was the greatest.

### 3.3. NaHS Inhibits PI3K Phosphorylation in dHL-60 Cells

**Figure 3:**
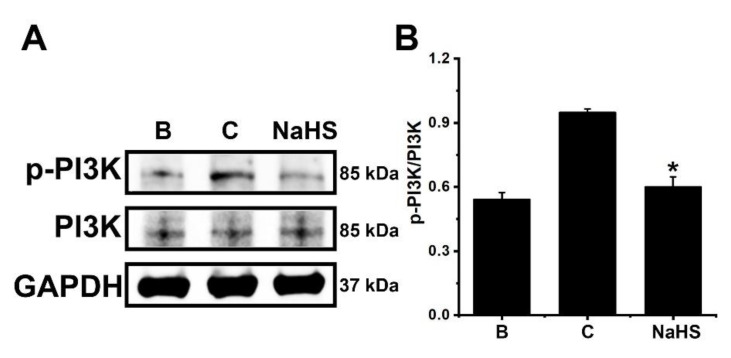
Inhibition of PI3K phosphorylation by NaHS in dHL-60 cells. (**A**) Cells (5 × 10^6^/mL) were pretreated with NaHS (1 mM) for 1 h, followed by GM-CSF-stimulation (5 ng/mL) for 15 min. (**B**) The protein levels were quantitated by densitometry. Blank (B) corresponds to PBS treated cells without GM-CSF stimulation, and control (C) corresponds to PBS treated cells stimulated by GM-CSF. Results are expressed as the mean ± SEM from three separate experiments. * *p* < 0.05, as compared with the PBS treated cells stimulated by GM-CSF.

To investigate the inhibitory mechanism of OSM suppression by NaHS, 1 mM of NaHS was pretreated in dHL-60 cells for 1 h, and then GM-CSF was treated for 15 min, since PI3K reached maximum phosphorylation 15 min after GM-CSF stimulation [23]. As demonstrated in previous work [23], GM-CSF stimulation led to increased phosphorylation of PI3K (Figure 3). However, this increased PI3K phosphorylation was reduced by pretreatment with NaHS (Figure 3).

### 3.4. NaHS Suppresses Akt Phosphorylation in dHL-60 Cells

**Figure 4:**
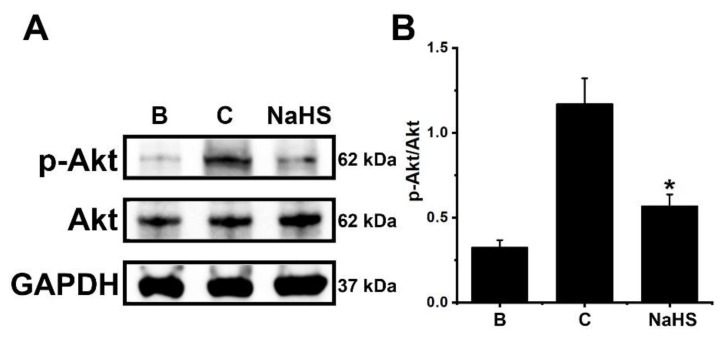
Inhibition of Akt phosphorylation by NaHS in dHL-60 cells. (**A**) Cells (5 × 10^6^/mL) were pretreated with NaHS (1 mM) for 1 h, followed by GM-CSF-stimulation (5 ng/mL) for 30 min. (**B**) The protein levels were quantitated by densitometry. Blank (B) corresponds to PBS treated cells without GM-CSF stimulation, and control (C) corresponds to PBS treated cells stimulated by GM-CSF. Results are expressed as the mean ± SEM from three separate experiments. * *p* < 0.05, as compared with the PBS treated cells stimulated by GM-CSF.

To study the inhibitory mechanism of OSM repression by NaHS, 1 mM of NaHS was pretreated in dHL-60 cells for 1 h, and then GM-CSF was treated for 30 min. As demonstrated elsewhere [23], GM-CSF stimulation led to increased phosphorylation of Akt (Figure 4). However, this elevated phosphorylation of Akt was reversed by pretreatment with NaHS (Figure 4). The Akt phosphorylation was lowered by pretreatment with a PI3K inhibitor, suggesting that Akt is a downstream factor of PI3K (Figure S4A). 

### 3.5. NaHS Downregulates NF-κB Phosphorylation in dHL-60 Cells

**Figure 5:**
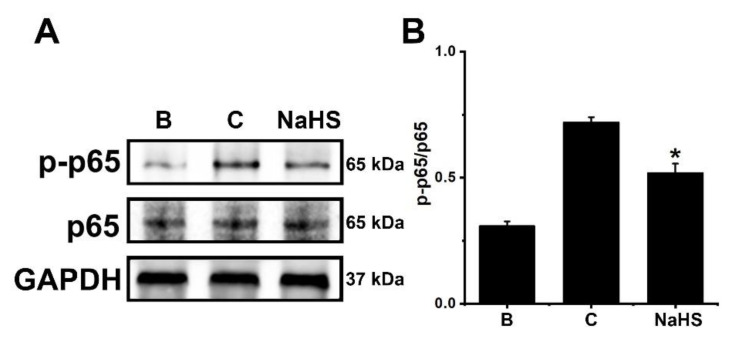
Inhibition of NF-κB phosphorylation by NaHS in dHL-60 cells. (**A**) Cells (5 × 10^6^/mL) were pretreated with NaHS (1 mM) for 1 h, followed by GM-CSF-stimulation (5 ng/mL) for 30 min. (**B**) The protein levels were quantitated by densitometry. Blank (B) corresponds to PBS treated cells without GM-CSF stimulation, and control (C) corresponds to PBS treated cells stimulated by GM-CSF. Results are expressed as the mean ± SEM from three separate experiments. * *p* < 0.05, as compared with the PBS treated cells stimulated by GM-CSF.

To identify the inhibitory mechanism of OSM downregulation by the NaHS, 1 mM of NaHS was pretreated in dHL-60 cells for 1 h, and then GM-CSF was treated for 30 min. As per previous work [23], upregulated phosphorylation of NF-κB resulted from GM-CSF stimulation (Figure 5). However, this elevated phosphorylation of NF-κB was abated by pretreatment with NaHS (Figure 5). The NF-κB phosphorylation was diminished by pretreatment with an Akt inhibitor, suggesting that NF-κB is a downstream factor of Akt (Figure S4B). 

### 3.6. NaHS Decreases p-NF-κB and OSM Immunofluorescence Staining in dHL-60 Cells 

**Figure 6:**
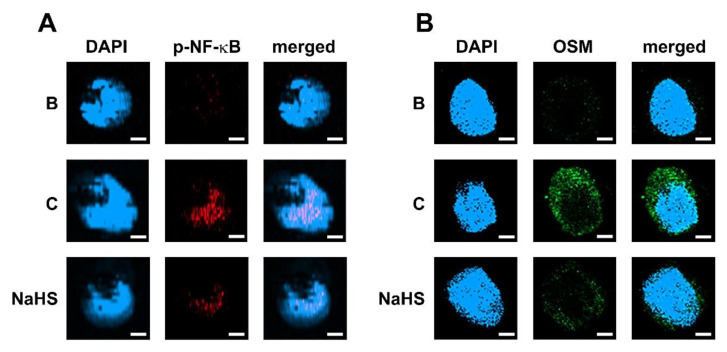
Inhibition of p-NF-κB and OSM immunofluorescence staining by NaHS in dHL-60 cells. (**A**) Cells (1 × 10^6^/mL) were pretreated with NaHS (1 mM) for 1 h, followed by GM-CSF stimulation (5 ng/mL) for 30 min; fluorescence microscope images stained with anti-p-p65 antibodies. (**B**) Cells (1 × 10^6^/mL) were pretreated with NaHS (1 mM) for 1 h, followed by GM-CSF stimulation (5 ng/mL) for 3 h; fluorescence microscope images stained with anti-OSM antibodies. Blank (B) corresponds to PBS treated cells without GM-CSF stimulation, and control (C) corresponds to PBS treated cells stimulated by GM-CSF. Representative images were obtained from images performed in triplicate (scale bar = 20 μm).

To clarify the inhibitory mechanism of NaHS using immunofluorescence analysis, we performed immunofluorescence staining for p-NF-κB, which is an important and final factor of the PI3K/Akt/NF-κB signal pathway, in dHL-60 cells. An amount of 1 mM of NaHS was pretreated in dHL-60 cells for 1 h, and then GM-CSF was treated for 30 min. Increased p-NF-κB immunofluorescence staining resulted from GM-CSF stimulation, however, the increased p-NF-κB immunofluorescence staining was downregulated by pretreatment with NaHS (Figure 6A). To confirm the suppression of OSM by NaHS using immunofluorescence analysis, we performed immunofluorescence staining for OSM in dHL-60 cells. An amount of 1 mM of NaHS was pretreated in dHL-60 cells for 1 h and then GM-CSF was treated for 3 h, since OSM immunofluorescence staining was greatest 3 h after GM-CSF stimulation. While increased OSM immunofluorescence staining resulted from GM-CSF stimulation, the increased OSM immunofluorescence staining was downregulated by pretreatment with NaHS (Figure 6B). 

## 4. Discussion

Plenty of research has indicated that OSM is highly expressed in a variety of inflammatory disorders, including chronic rhinosinusitis and asthma [22,51,52]. One study reported that GM-CSF activation led to increased levels of OSM mRNA expression [53]. Additionally, lots of research has suggested that GM-CSF activation results in upregulation of OSM in human neutrophils [22,29,54,55]. In our previous work [23], our findings also revealed that GM-CSF activation led to elevated levels of OSM production and mRNA expression (Figure 1A and Figure 2). Increased levels of OSM production and mRNA expression were attenuated by pretreatment with NaHS (Figure 1A and Figure 2). In our previous work, stimulation with recombinant human OSM led to increased IL-1β secretion in human HaCaT keratinocytes, suggesting that OSM contributed to inflammatory reactions [23]. Furthermore, increased IL-1β expression of HaCaT cells resulted from culture of the HaCaT cells with conditioned medium from GM-CSF-stimulated dHL-60 cells [23]. Thus, decreased OSM expression by NaHS may, at least in part, alleviate inflammatory reactions in keratinocytes. Intranasal application of OSM protein showed increased inflammatory infiltrate as well as elevations in the levels of inflammatory cytokines and chemokines in mice [56], and OSM hypodermic injection led to exacerbated skin inflammation in mice [57]. Another study revealed increased inflammatory reactions in the pulmonary tissues of OSM-overexpressing mice [58]. In asthmatic subjects, OSM protein and mRNA have been shown to be highly expressed, while non-asthmatic subjects exhibited no expression of OSM [52]. In addition, OSM neutralization and OSM knockout mice showed downregulated inflammation in colonic tissues of mice [11]. Hence, we can presume that NaHS might be useful to treat inflammatory disorders by blockading of OSM. To have a convincing result that NaHS can regulate the expression of OSM, we used mast cell line HMC-1 cells. Stimulation of HMC-1 cells with PMA plus A23187 is known to induce an increase in various inflammatory factors, such as IL-1β, IL-6, IL-8, TNF-α, and TSLP [59,60,61]. However, there was no increase in OSM levels by PMA plus A23187 (Figure S2B). From our result (Figure S2B), we could assume that the main source of OSM in inflammatory conditions stems from neutrophil cells, not mast cells. 

In general, PI3K/Akt signal processes are important in the regulation of inflammatory reactions [24,25,26,27]. It is widely known that NF-κB plays a crucial role in inflammatory responses [28]. More specifically, PI3K/Akt/NF-κB signaling processes were found to be responsible for OSM production in osteoblasts [28]. As shown in our previous work [23], the PI3K/Akt/NF-κB signaling processes were also responsible for OSM production in dHL-60 cells. He and colleagues [62] reported that application of a PI3K inhibitor reduced mRNA expression and proteins of a variety of inflammatory factors, including IL-1β, IL-6, and TNF-α, in an experimental model. Furthermore, the blocking of the PI3K/Akt signal pathway led to an improvement in arthritis in a mouse model [63]. Treatment with wortmannin, LY-294002, and IC87114, which are excellent PI3K inhibitors, resulted in reduced airway hyperresponsiveness and inflammatory responses in mice [64,65]. Bao and colleagues [66] reported that suppressed lung tissue inflammation resulted from the application of an Akt inhibitor in a mouse model. In addition, NF-κB inhibition downregulated pulmonary inflammation in a murine model of asthma [66,67]. The results of the present work showed that pretreatment with NaHS deceased phosphorylation of PI3K, Akt, and NF-κB (Figure 3, Figure 4 and Figure 5). Co-treatment of NaHS with a PI3K inhibitor as well as NaHS with a NF-κB inhibitor showed a synergy effect in the suppression of OSM mRNA expression in dHL-60 cells (Figure S3). It is therefore possible to assume that the PI3K/Akt/NF-κB signal cascade may, at least in part, affect OSM regulation via NaHS in dHL-60 cells. 

## 5. Conclusions

**Figure 7:**
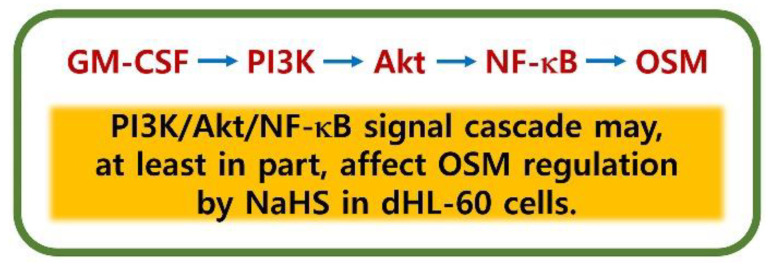
Schematic diagram of OSM suppression by NaHS.

We demonstrated that NaHS inhibited OSM expression and phosphorylation of PI3K, Akt, and NF-κB in dHL-60 cells (Figure 7). Our findings indicate that H_2_S might be useful in the treatment of inflammatory disorders. 

## Data Availability

Data is contained within the article and supplementary material.

## Supplementary Materials

The following supporting information can be downloaded at: https://www.mdpi.com/article/10.3390/antiox12020417/s1, Figure S1: Neutrophil markers in HL-60 cells (DMSO: -) and differentiated HL-60 cells (DMSO: +). Figure S2: The OSM levels in dHL-60 cells and HMC-1 cells. Figure S3: OSM mRNA expression in dHL-60 cells. Figure S4: Phosphorylated levels of Akt and p65 in dHL-60 cells.
