# Supplementary material for: Hydrogen Sulfide Downregulates Oncostatin M Expression via PI3K/Akt/NF-κB Signaling Processes in Neutrophil-like Differentiated HL-60 Cells
Source: Antioxidants (Basel). 2023 Feb 8;12(2):417. doi: 10.3390/antiox12020417 (PMC9952767; doi:10.3390/antiox12020417)
Supplement: Supplementary file 1 [file antioxidants-12-00417-s001.zip › antioxidants-2099055-supplementary.pdf]

antioxidants

MDPI

Supplementary

Hydrogen Sulfide Downregulates Oncostatin M Expression via PI3K/Akt/NF-κB Signaling Processes in Neutrophil-like Differentiated HL-60 Cells

Na-Ra Han [1,2], Seong-Gyu Ko [2,3], Hi-Joon Park [4] and Phil-Dong Moon [5,*]

[1] College of Korean Medicine, Kyung Hee University, Seoul, Republic of Korea
[2] Korean Medicine-Based Drug Repositioning Cancer Research Center, College of Korean Medicine, Kyung Hee University, Seoul, Republic of Korea
[3] Department of Preventive Medicine, College of Korean Medicine, Kyung Hee University, Seoul, Republic of Korea
[4] Department of Anatomy & Information Sciences, College of Korean Medicine, Kyung Hee University, Seoul, Republic of Korea
[5] Center for Converging Humanities, Kyung Hee University, Seoul, Republic of Korea
* Correspondence: pdmoon@khu.ac.kr; Tel.: +82-2-961-0897

Results

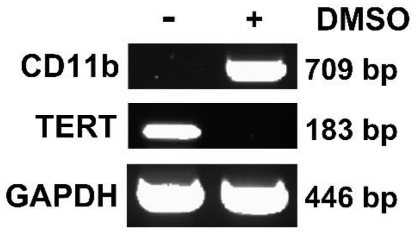

Figure S1. Neutrophil markers in HL-60 cells (DMSO: -) and differentiated HL-60 cells (DMSO: +). CD11b is known to be expressed in neutrophil-differentiated cells but not in undifferentiated ones. Conversely, expression of TERT is attenuated during differentiation.

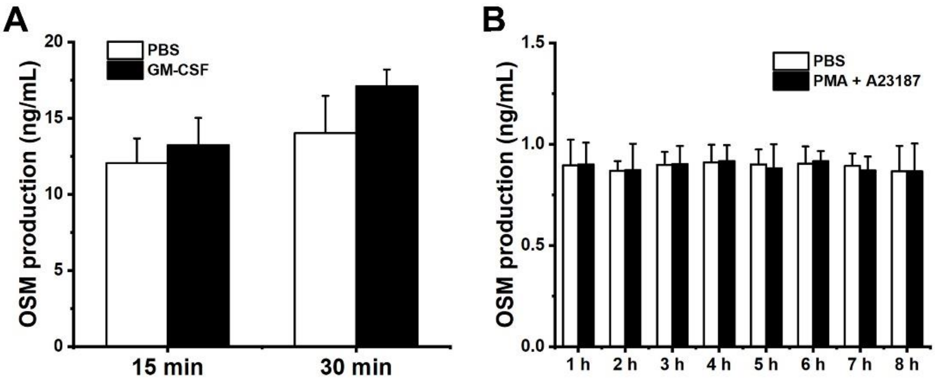

Figure S2. The OSM levels in dHL-60 cells and HMC-1 cells. (**A**) dHL-60 cells (5×10^5/mL) were stimulated with GM-CSF (5 ng/mL) for the indicated time. (**B**) HMC-1 cells (5×10^5/mL) were stimulated with phorbol myristate acetate (0.05 µM) plus A23187 (1 µM) for the indicated time. Results are expressed as the mean ± SEM.

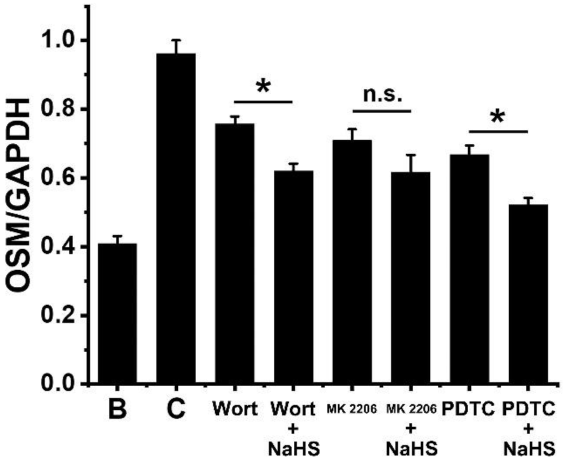

Figure S3. OSM mRNA expression in dHL-60 cells. Cells (1 × 10^6/mL) were treated with each inhibitor or co-treated with each inhibitor and NaHS (1 mM) for 1 h, followed by GM-CSF-stimulation (5 ng/mL) for 30 min. Blank (B) corresponds to PBS treated cells without GM-CSF stimulation, and control (C) corresponds to PBS treated cells stimulated by GM-CSF. Wort, 50 μM of wortmannin (PI3K inhibitor) treated cells; MK 2206, 50 μM of MK 2206 (Akt inhibitor) treated cells; PDTC, 100 μM of PDTC (NF-κB inhibitor) treated cells; n.s., not significant. Results are expressed as the mean ± SEM. *$p < 0.05$, as compared with each inhibitor treated cells stimulated by GM-CSF.

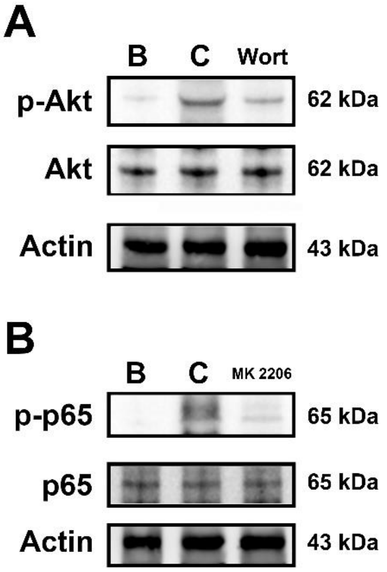

Figure S4. Phosphorylated levels of Akt and p65 in dHL-60 cells. (**A**) The dHL-60 cells (5 × 10^6/mL) were pretreated with PI3K inhibitor (wortmannin, Wort, 50 μM) for 1 h, followed by GM-CSF-stimulation (5 ng/mL) for 30 min. (**B**) The dHL-60 cells (5 × 10^6/mL) were pretreated with Akt inhibitor (MK 2206, 50 μM) for 1 h, followed by GM-CSF-stimulation (5 ng/mL) for 30 min. Blank (B) corresponds to PBS treated cells without GM-CSF stimulation, and control (C) corresponds to PBS treated cells stimulated by GM-CSF.

Materials and Methods

Cell Viability

dHL-60 cells (1 × 10^5/mL) were seeded in 24-well plate and pretreated with NaHS or PBS for 1 h, and then stimulated with GM-CSF (5 ng/mL) for 4 h. The cells were incubated with 3-(4,5-dimethylthiazol-2-yl)-2,5-diphenyltetrazolium bromide (MTT, Sigma-Aldrich Co.) solution at 37 °C for 4 h. Next, we added 1 mL of dimethyl sulfoxide (DMSO) to dissolve the MTT formazan, and transferred 100 μL of supernatant into a new 96-well microplate. A microplate reader (540 nm, Versa Max, Molecular Devices, Sunnyvale, CA, USA) was used to measure the absorbance of formazan dissolved in DMSO.

OSM Measurement

dHL-60 cells (5 × 10^5/mL) were seeded in 24-well plate and pretreated with NaHS or PBS for 1 h, and then stimulated with GM-CSF (5 ng/mL) for 4 h. OSM levels were assessed by means of an enzyme-linked immunosorbent assay. The capture antibody (R&D system Inc., Minneapolis, MN, USA) was pre-coated in a 96-well plate. Phosphate-buffered saline (PBS) containing 10% FBS was added to block the plate for 2 h. After washing the plate by means of PBS containing Tween 20 (PBST), cell supernatants were added into the plate for 2 h. After washing the plate with PBST, the plate was treated with biotinylated detection antibody (R&D system Inc.) for 2 h and then incubated with avidin-conjugated to horseradish peroxidase (Sigma-Aldrich Co.) for 30 min. Absorbance by TMB substrate (BD Pharmingen, San Jose, CA, USA) was measured by a microplate reader (405 nm, Versa Max).

Real-Time Quantitative PCR

dHL-60 cells (1 × 10^6/mL) were seeded in 6-well plate and pretreated with NaHS or PBS for 1 h, and then stimulated with GM-CSF (5 ng/mL) for 30 min. The harvested cells were used to isolate total RNA by means of an RNA extraction reagent (iNtRON, Seongnam, Republic of Korea). The first-strand cDNA from total RNA was synthesized with cDNA synthesis reagents (Bioneer, Daejeon, Korea). The following designed primers were used for the real time PCR (Applied Biosystems, Foster City, CA, USA) by using Power SYBR® Green Master Mix (Applied Biosystems): OSM: 5′-GCTCACACAGAGGACGCTG-3′, 5′-GGAGCACGCGGTACTCTTTC-3′; GAPDH: 5′-TCGACAGTCAGCCG-CATCTTCTTT-3′, 5′-ACCAAATCCGTTGACTCCGACCTT-3′. The relative expression of mRNA for OSM was normalized by GAPDH and measured by using $2^{-\Delta\Delta Ct}$ method.

Reverse Transcription-PCR

Easy-BLUE™ RNA extraction kit (iNtRON Biotech, Republic of Korea) was used to isolate the total RNA from dHL-60 cells in accordance with the manufacturer's specifications. The concentrations of total RNA in the final elutes were determined by a spectrophotometer. Total RNA (1 μg) was heated at 65°C for 10 min and then chilled on ice. Each sample was reverse-transcribed to cDNA for 90 min at 37°C using a cDNA synthesis kit (Amersham Pharmacia Biotech, Piscataway, NJ, USA). The PCR was performed with the following primers for human CD11b (5′-TGG CTC TCA GAG TCC TTC TGT-3′; 5′-TCT CGT ACC ACT TTG CGG AT-3′) and human TERT (5′-AGA GTG TCT GGA GCA AGT TGC-3′; 5′-CGT AGT CCA TGT TCA CAA TCG-3′). GAPDH (5′-CAA AAG GGT CAT CAT CTC TG-3′; 5′-CCT GCT TCA CCA CCT TCT TG-3′) was used to verify if equal amounts of RNA were used for reverse transcription and PCR amplification under different experimental conditions. The annealing temperature was 60°C for CD11b and TERT, 62°C for GAPDH. Amplified fragment sizes for CD11b, TERT, and GAPDH were 709 bp, 183 bp, and 446 bp, respectively. Products were electrophoresed on a 1.5% agarose gel and visualized by staining with ethidium bromide.

Western Blot Analysis

dHL-60 cells (5 × 10^6/mL) were seeded in 60 mm dish and pretreated with NaHS or PBS for 1 h, and then stimulated with GM-CSF (5 ng/mL) for 15 min (PI3K) or 30 min (Akt) or 30 min (NF-κB). An ice-cold cell lysis buffer (Sigma-Aldrich Co.) was used to lyse the harvested cells. Cell extracts were prepared with sampling buffer (Laemmli's 2×, ELPISBI-OTECH. INC., Daejeon, Korea) and heated at 95 °C for 5 min. Proteins were subjected to electrophoresis using 10% - 15% gel containing sodium dodecyl sulfate and transferred to nitrocellulose membranes (Amersham™, Chicago IL, USA). PBST containing 5% bovine serum albumin (Sigma-Aldrich Co.) was used to block the membranes afterwards relevant primary antibodies (phosphorylated (p)-PI3K, Cell Signaling Technology, Danvers, MA, USA; PI3K, p-Akt, Akt, p-p65, p65, and GAPDH, Santa Cruz Biotechnology, Santa Cruz, CA, USA) were used. Peroxidase-conjugated secondary antibodies (Santa Cruz Biotechnology) were added for incubation of the membranes for 1 h at room temperature after washing with PBST. Specific bands were detected by an enhanced chemiluminescence solution (DoGenBio Co., Seoul, Korea). Band intensities were calculated with ImageJ program (National health institute, Bethesda, MD, USA).

Immunofluorescence Analysis

dHL-60 cells (1 × 10^6/mL) were seeded in 60 mm dish and pretreated with NaHS or PBS for 1 h, and then stimulated with GM-CSF (5 ng/mL) for 30 min. dHL-60 cells were fixed with 4% paraformaldehyde, permeabilized in 0.2% Triton X-100, and incubated with a blocking buffer to reduce nonspecific binding. The cells were incubated with the primary antibodies (anti-p-p65 and anti-OSM), followed by incubation with Alexa Fluor® conjugated secondary antibodies (Alexa Fluor® 647 for p-p65k, Alexa Fluor® 488 for OSM, Abcam, Cambridge, MA, USA) at room temperature. For

nuclear staining, 4′ ,6-diamidino-2-phenylindole (DAPI) was used. Samples were visualized under a confocal laser-scanning microscope (Carl Zeiss, Oberkochen, Germany).
